# Image Alignment for Tomography Reconstruction from Synchrotron X-Ray Microscopic Images

**DOI:** 10.1371/journal.pone.0084675

**Published:** 2014-01-09

**Authors:** Chang-Chieh Cheng, Chia-Chi Chien, Hsiang-Hsin Chen, Yeukuang Hwu, Yu-Tai Ching

**Affiliations:** 1 Department of Computer Science, National Chiao Tung University, Hsinchu, Taiwan; 2 Institute of Physics, Academia Sinica, Taipei, Taiwan; 3 Department of Engineering and System Science, National Tsing Hua University, Hsinchu, Taiwan; 4 Ian Wark Research Institute, University of South Australia, Adelaide, Australia; 5 Institute of Biomedical Engineering, National Chiao Tung University, Hsinchu, Taiwan; Universidad de Castilla-La Mancha, Spain

## Abstract

A synchrotron X-ray microscope is a powerful imaging apparatus for taking high-resolution and high-contrast X-ray images of nanoscale objects. A sufficient number of X-ray projection images from different angles is required for constructing 3D volume images of an object. Because a synchrotron light source is immobile, a rotational object holder is required for tomography. At a resolution of 10 nm per pixel, the vibration of the holder caused by rotating the object cannot be disregarded if tomographic images are to be reconstructed accurately. This paper presents a computer method to compensate for the vibration of the rotational holder by aligning neighboring X-ray images. This alignment process involves two steps. The first step is to match the “projected feature points” in the sequence of images. The matched projected feature points in the 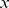-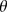 plane should form a set of sine-shaped loci. The second step is to fit the loci to a set of sine waves to compute the parameters required for alignment. The experimental results show that the proposed method outperforms two previously proposed methods, Xradia and SPIDER. The developed software system can be downloaded from the URL, http://www.cs.nctu.edu.tw/~chengchc/SCTA or http://goo.gl/s4AMx.

## Introduction

Synchrotron X-rays possess several unique characteristics including high intensity, straight-line beam traveling, and low scattering [1], which can be used to develop an X-ray microscope [2]. Researchers have developed many synchrotron X-ray microscopy techniques for various purposes including angiography [3], X-ray lithography for nanofabrication [4], and cell tomography[5]–[7]. This study investigated a problem arising from 3D tomography reconstruction when the pixel size is in the nanoscale.

**Figure 1:**
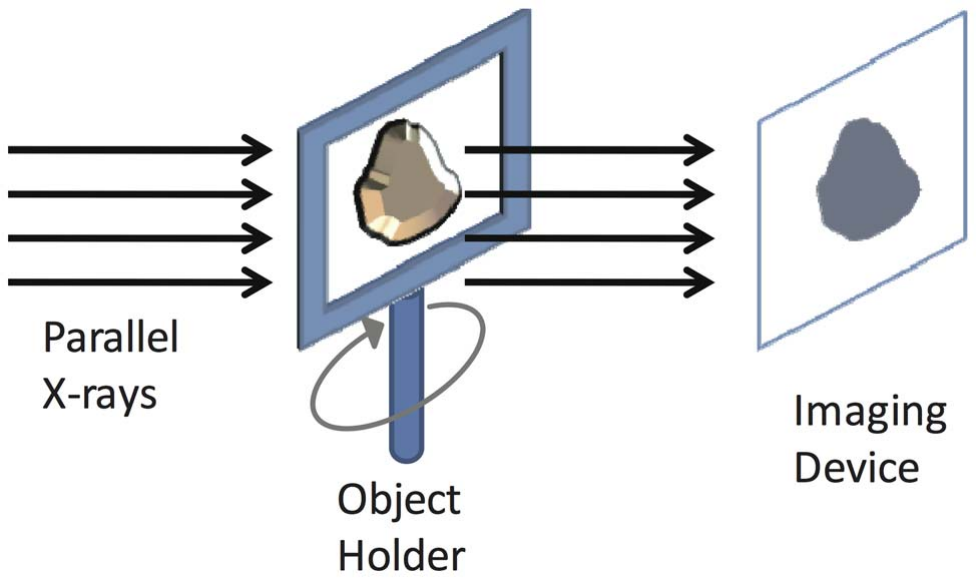
View of the rotational holder for acquiring the projection images for reconstructing the 3D volume images using Synchrotron light source and for correcting the horizontal alignment due to vibration below 10 nm.

Previous research has demonstrated that a series of X-ray projections around an object can be used to reconstruct 3D volume data by using the appropriate reconstruction algorithms [8]–[11]. When the light source is a synchrotron X-ray, it cannot rotate around an object. Therefore, a rotatable object-holder was designed to hold an object to enable acquiring a series of projections from different angles while rotating the object (Fig. 1). The problem of mechanical imprecision arises when the resolution increases to a certain level, such as that required for cell tomography. When the pixel size is approximately 10 nm, a slight mechanical vibration can hinder accurate reconstruction. In the cell tomography experience of the authors, the pixel size of image is 11.78 nm. Rotating the object holder can cause a 5 to 30 pixel difference in position because of the mechanical instability. Although the position of the object holder can be calibrated during image acquisition, the calibration process can take an unacceptably long time, causing the object to receive excessive X-rays. The TXM controller provided by Xradia (hereinafter called “Xradia”) was designed to solve the misalignment problem. Unfortunately, manual adjustments are generally required to obtain satisfactory tomography reconstruction. A similar problem also exists in electron microscopic tomography. Using the cross-correlation function to align the electron microscopic images is a common solution to this problem [12]. A software system, “SPIDER”, was implemented based on the cross-correlation function[13]. However, the cross-correlation function alignment does not considering the projection model, thus limiting the quality of tomography reconstruction.

This study presents a feature-based alignment approach for calibrating the displacement caused by mechanical vibration. Because a synchrotron X-ray is a parallel beam projection, the resulting displacement can be decomposed into vertical and horizontal displacements. The proposed method aligns the images in the vertical direction by direct image alignment. Calibrating the images in the horizontal direction is more complex than that in the vertical direction. In addition to matching feature points, the matched feature points must form sine-wave shaped loci. This study proposes approximating the loci of the matched feature points in the 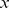-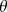 coordinate system according to sine waves by using the least square curve fitting. The deviation between the loci and the sine waves provides information for horizontal calibration.

The remainder of this paper is organized as follows. The Results section presents the results. The Methods section presents the details of the proposed methods including feature detection, feature tracking, and the alignment method. Finally, the Discussion section presents a summary and discussion.

## Results

The proposed algorithm was used to align projection images and then reconstruct the 3D volume data of HeLa cells from X-ray projections. To verify the correctness of the alignment algorithm, a phantom data set was used to simulate the HeLa cell stained using the gold nanoparticles. The following subsections present the construction details and results obtained.

### The Phantom

**Figure 2:**
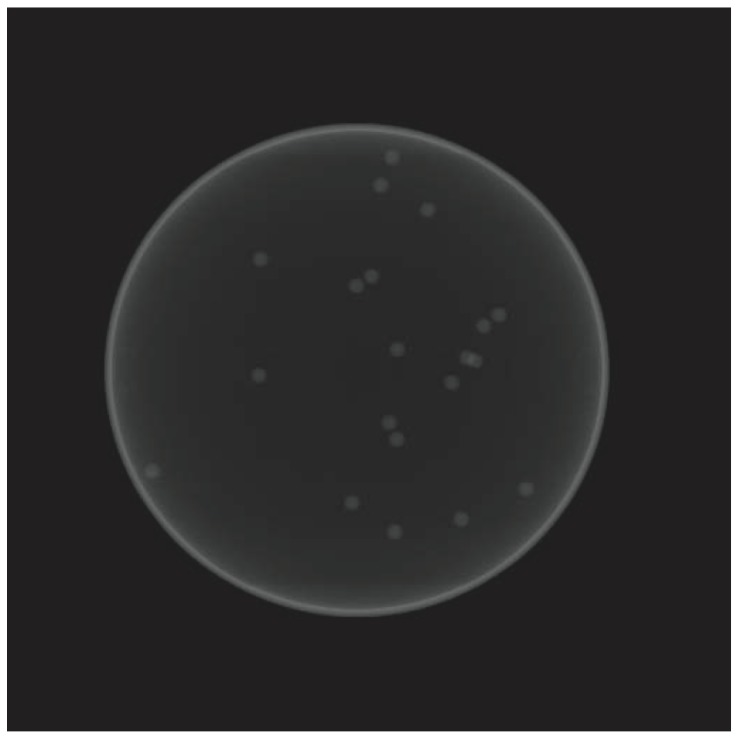
The simulated X-ray image of the phantom data. 180 emulated X-ray images were generated. The size of each image is 512×512 pixels, the rotation angle between two consecutive images is 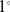, and the ranges of the vertical and horizontal errors are 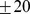 pixels.

A volume datum containing 20 imitative gold nanoparticles was constructed, and X-ray projections of the shifted volume were generated to simulate machine vibrations. Fig. 2 shows the simulated X-ray image of the phantom cell, in which 180 projections of 512×512 pixels images were generated. The volume rotated is 1° between successive projections. Before each projection, the volume was shifted in the vertical and horizontal directions to simulate the mechanical imprecision of the object holder. The amount of displacement was determined according to a random number uniformly distributed over the range ±20 pixels.

**Figure 3:**
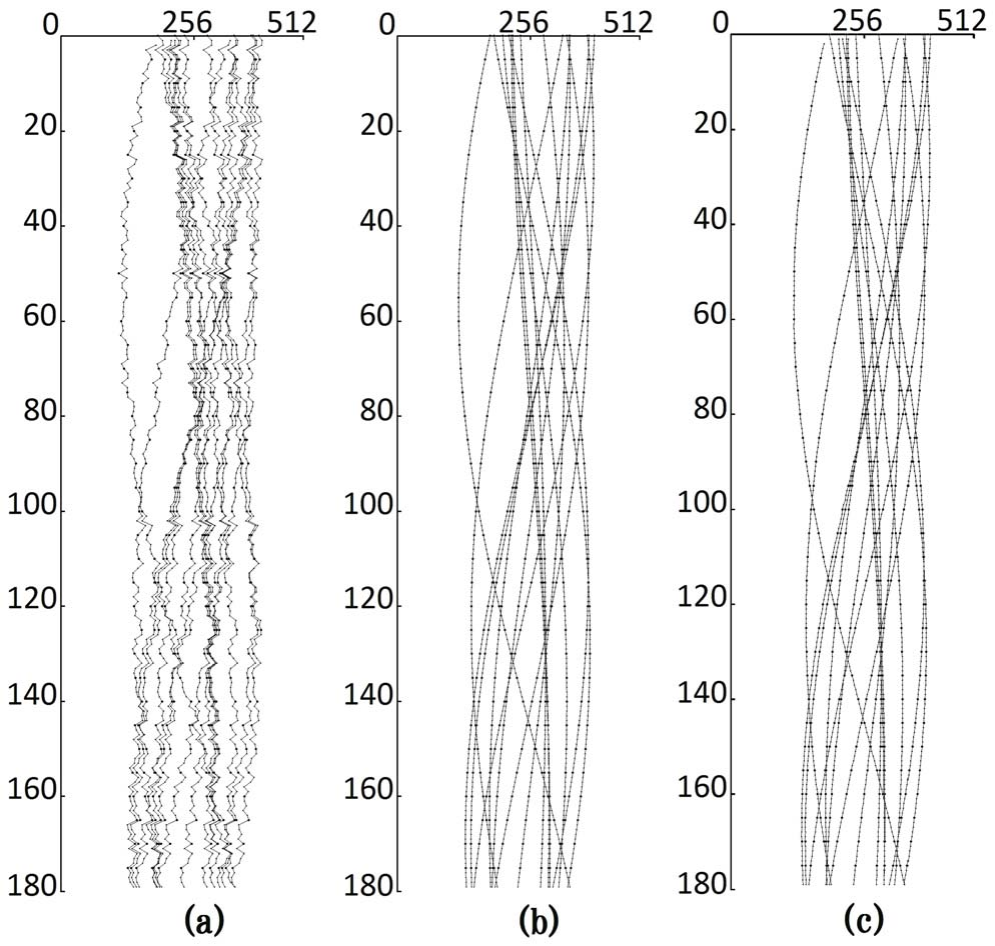
Feature loci of the phantom data: (a) loci of 16 projected feature points before horizontal alignment; (b) best-fit sine waves; and (c) the aligned loci.

The proposed alignment algorithm was used to calibrate the images, which were first aligned in the vertical direction. The detection and matching methods of projected feature points were then applied to determine 16 feature points. The locus of the horizontal position of a feature point over various angles should form a sine-shaped curve. Fig. 3(a) shows the loci of the 16 points. This figure is in the 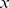-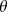 coordinate system: the vertical axis represents the rotation angle, and the horizontal axis represents the horizontal position of the projected feature point. The loci are not smooth because of horizontal displacement. The most suitable sine waves to fit the loci (Fig. 3(b)) were then calculated, and the displacement of the feature points in the horizontal position were estimated (Fig. 3(c)). The filtered back-projection (FBP) algorithm was then used to reconstruct the 3D volume data based on the aligned X-ray images [14].

**Figure 4:**
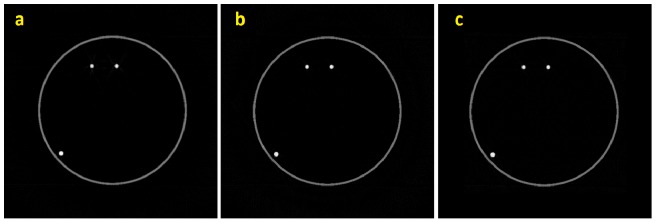
One slice in the phantom data: (a) original image; (b) result of proposed method; and (c) the result of SPIDER.

To verify the accuracy of the proposed alignment method, the positions and diameters of the spherical particles in the reconstructed images were compared with the original volume data, which included 20 spherical particles, and the diameter of each particle was 12 voxels. There were 20 particles found in the reconstructed volume. The average errors of the particle center and diameter were found to be 0.72 and 0.03 voxels, respectively. The reconstructed volume is close to the original volume. Fig. 4(a) shows a slice in the original volume data. Fig. 4(b) shows the tomography results of the same slice obtained using the proposed alignment method. For comparing the results of the proposed method with the SPIDER, the same phantom image was reconstructed using SPIDER. Fig. 4(c) shows the tomography reconstructed results using SPIDER.

The mean squared error (MSE) of the foreground voxels between the original volume data and the reconstructed volume was also calculated. A voxel was classified as a foreground voxel when its intensity exceeded a given intensity threshold. The MSE between the original volume and the reconstructed volume from the unaligned volume was found to be 0.029. The reconstructed volume obtained from the images aligned using SPIDER has an MSE of 0.002. For the volume reconstructed from the images aligned using the proposed method, the MSE was found to reduce to 0.0005. Because Xradia does not recognize the file format of the phantom images, the proposed method was not compared with Xradia.

### HeLa Cells

**Figure 5:**
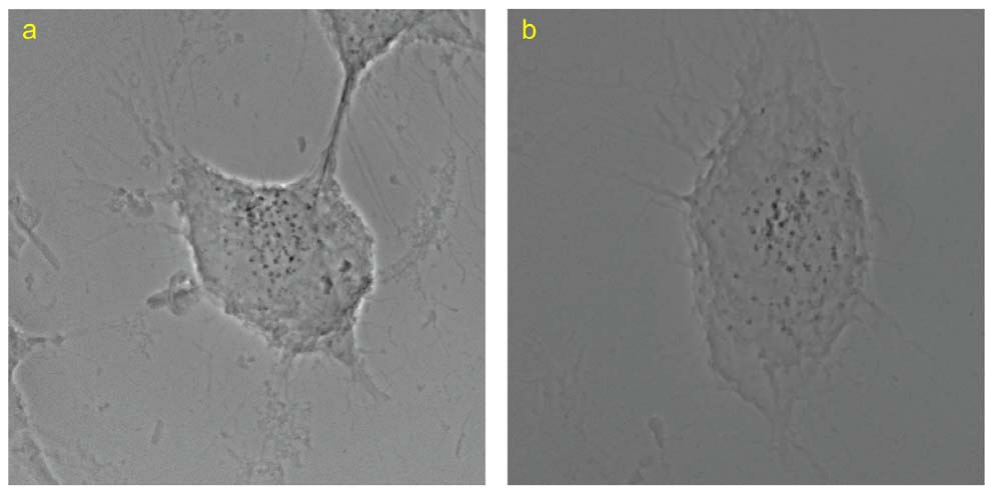
X-ray projection images of HeLa cells. 140 synchrotron X-ray projection images were acquired for HeLa1 and HeLa2. The size of each image is 1024×1024 pixels, and the rotation angle between two projection images is 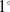. (a) HeLa1, and (b) HeLa2.

**Figure 6:**
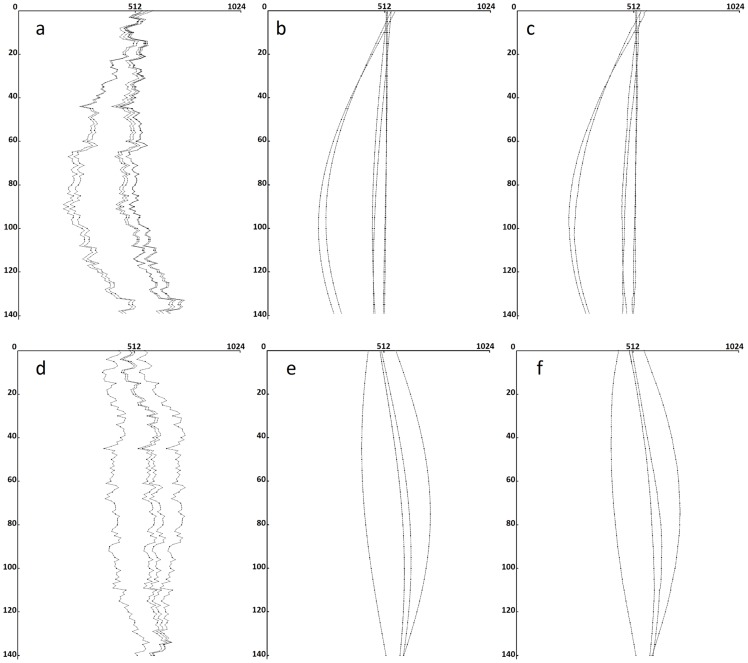
The loci of the reliable projected feature points of HeLa1 and HeLa2. (a) There were six reliable feature loci found in the acquired images of HeLa1. (b) Most suitable sine waves of HeLa1, and (c) aligned loci of HeLa1. (d) There were four reliable feature loci identified in the acquired images of HeLa2. (e) Best-fit sine waves of HeLa2, and (f) aligned loci of HeLa2.

Figs. 5(a) and (b) separately show the projection images of two HeLa cells. The first HeLa cell, named HeLa1, contained 84 identified projected feature points. Six reliable feature points were selected from the identified projected feature points. Fig. 6(a) shows the loci of the selected points in the 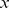-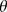 coordinate system. Fig. 6(b) shows the sine waves that fit the loci most effectively. The projection images of HeLa1 are aligned according to the fitted sine waves, and Fig. 6(c) shows the loci of the projected feature points after alignment. In the second HeLa cell, HeLa2, four reliable features were selected from 89 identified projected feature points, and Fig. 6(d) shows the loci. Fig. 6(e) shows the sine waves that fit the loci. Fig. 6(f) shows the loci of the projected feature points after alignment. To compare the different alignment methods, SPIDER and Xradia were also applied to align the projection images of HeLa1 and HeLa2. The same FBP algorithm was then applied to reconstruct the volumes from the aligned images.

**Figure 7:**
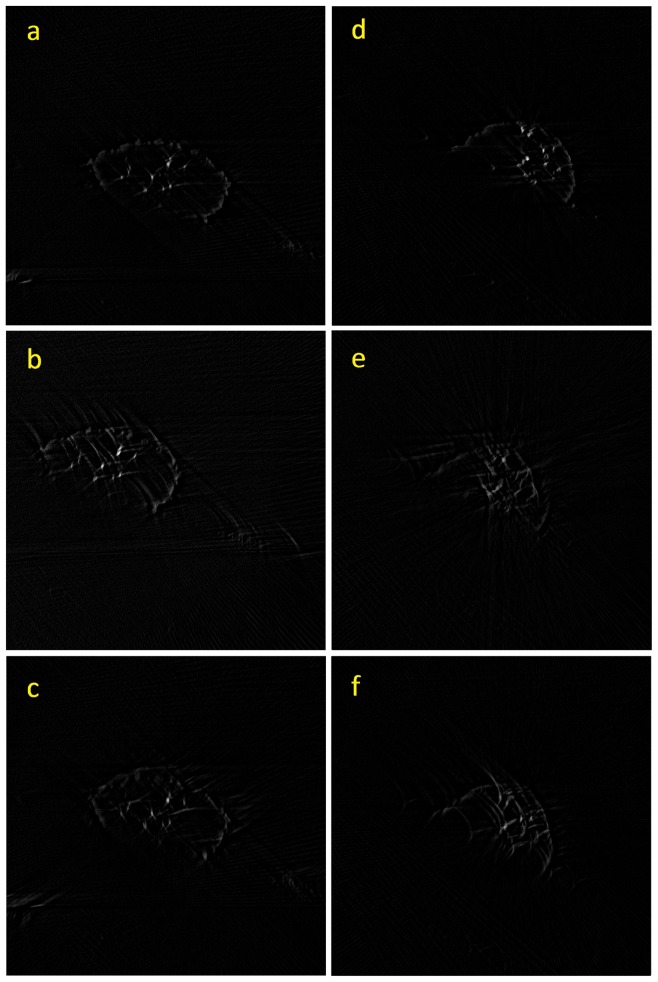
One slice in the reconstructed tomographic images of HeLa1 and HeLa2. (a), (b), and (c) are the same slice in the tomographic images of HeLa1: (a) result of proposed method; (b) result of SPIDER, and (c) result of Xradia. (d), (e), and (f) are the same slice in the tomographic images of HeLa2: (a) result of proposed method; (b) result of SPIDER, and (c) result of Xradia.

**Figure 8:**
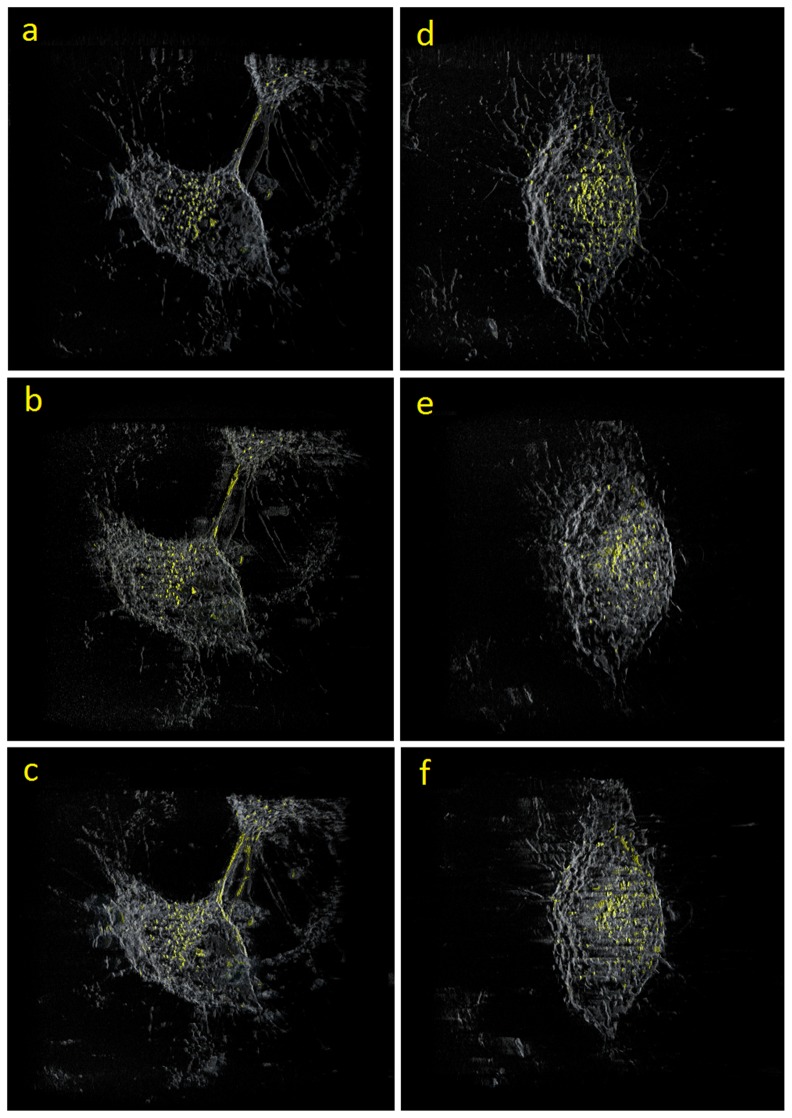
The 3D volume rendering of the reconstructed volume (a), (b), and (c) are HeLa1; (d), (e), and (f) are HeLa2: (a) and (d) Results of proposed method; (b) and (e) results of SPIDER, and (c) and (f) results of Xradia.

Slices in the reconstructed volumes of HeLa1 and HeLa2 are shown in Figs. 7(a), (b), and (c) and Figs. 7(d), (e), and (f) respectively. Figs. 7 (a) and (d) were obtained using the proposed method, Figs. 7 (b) and (e) were obtained using SPIDER, and Fig. 7 (c, f) were obtained using Xradia. As shown in Fig. 7, the results of the proposed method were more favorable than those of SPIDER and Xradia. Comparing the results of the SPIDER and Xradia, the proposed method exhibits most well-defined membrane structures with least amount of artifacts, which is evident from 7(a) and (d). The texture-based volume rendering algorithm [15] was used to produce a 3D image of the volume data. Figs. 8 (a), (b), and (c) show the volume-rendering results of HeLa1, and Figs. 8 (d), (e), and (f) show the volume-rendering results of HeLa2. Figs. 8 (a) and (d) show the results of the proposed method, Figs. 8 (b) and (e) show the results of SPIDER, and Figs. 8 (c) and (f) show the results of Xradia. The gold nanoparticles in Figs. 8 (a) and (d) are clearly shown, thereby enabling the evaluation of the location, the size, and the amount of the particles. The cell membranes can also be visualized in the rendered image, helping the user identify the geometry of the cells.

**Figure 9:**
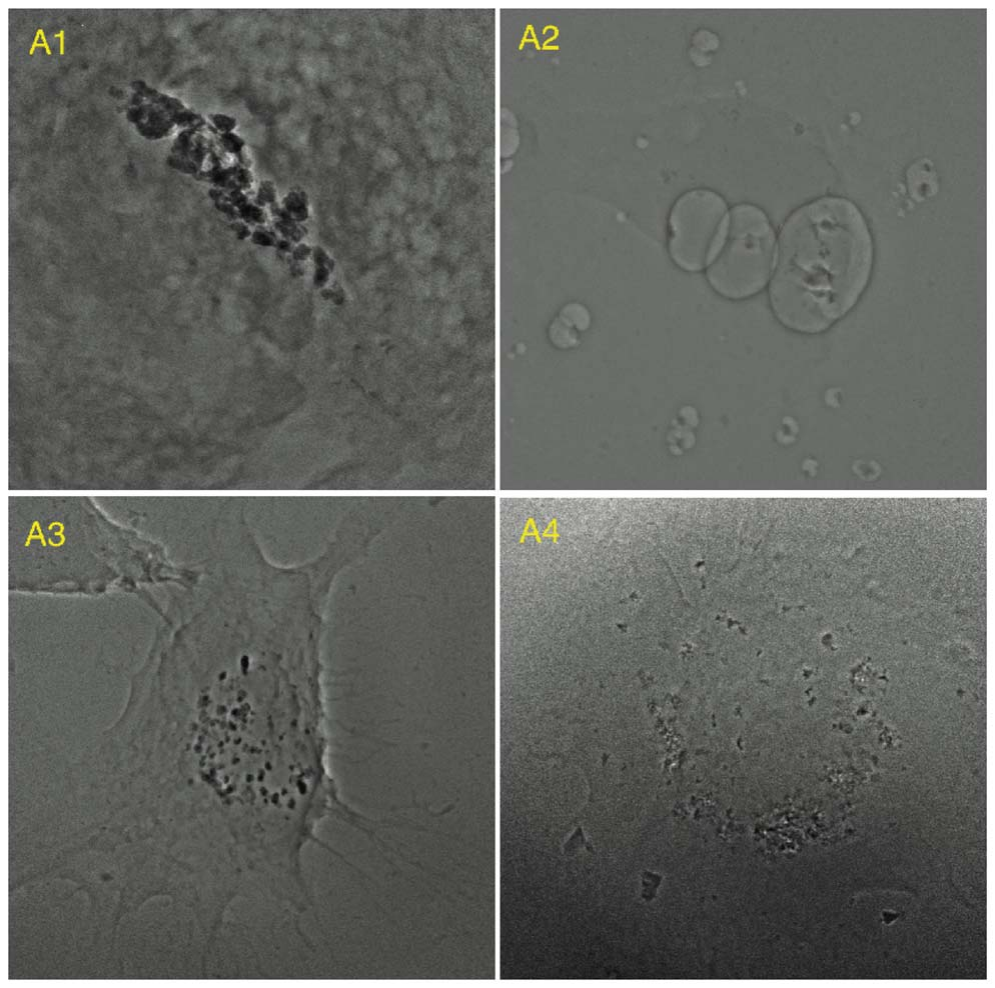
Eight examples of successful alignment (1).

**Figure 10:**
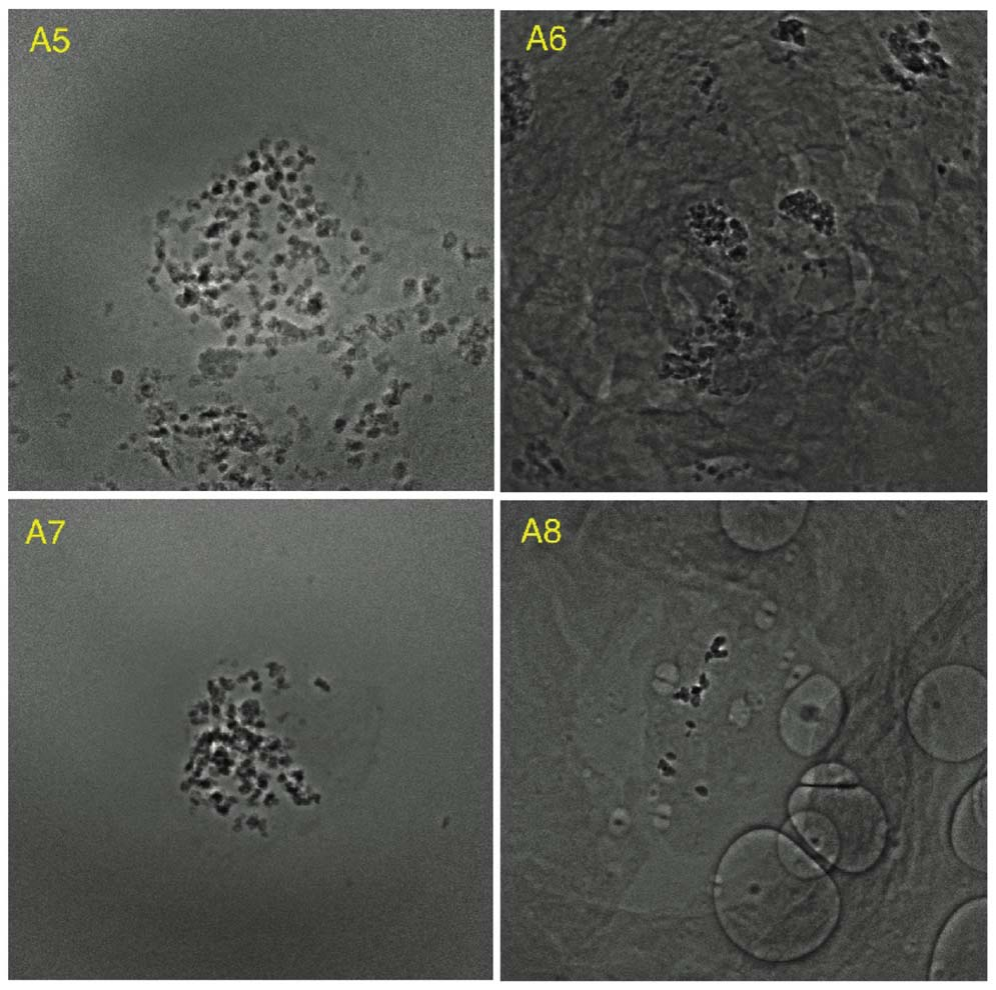
Eight examples of successful alignment (2).

**Figure 11:**
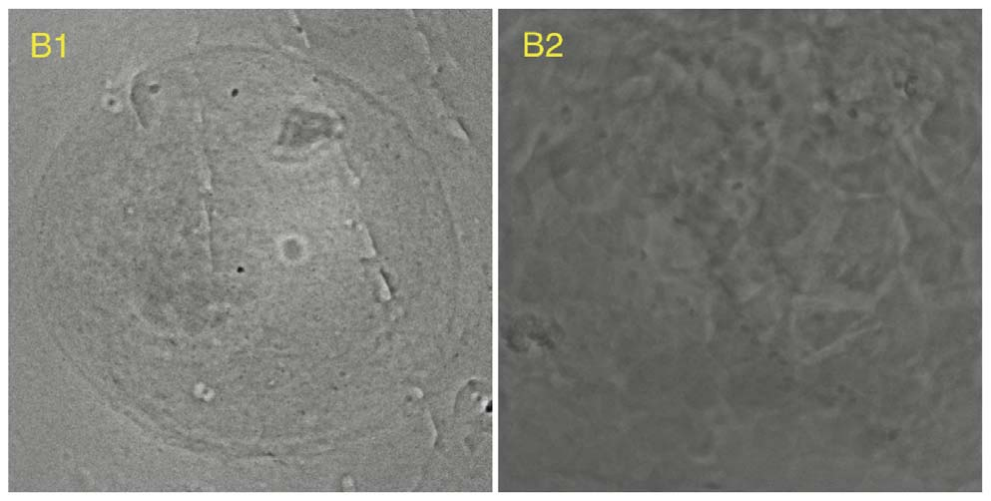
Two examples of unsuccessful alignment.

The proposed algorithm was applied to process 10 other X-ray image sets of HeLa cells. Among these 10 image sets, eight were successfully reconstructed (A1–A8, Figs. 9 and 10) except the other two (B1 and B2, Fig. 11). SPIDER and Xradia were also applied to the same sets of image data. Neither SPIDER nor Xradia could align the images in B1 and B2 for reconstruction. Specifically, SPIDER was effective for A3, A5, and A6; Xradia was effective for A1, A2, A5, A7, and A8. Although the reconstructions could be completed, comparing to our results, the artifacts produced due to the misalignment are more apparent.

Table 1 lists the experiments conducted in this study including the phantom, HeLa1, HeLa2, and the other 10 HeLa cells, as well as the computing time required. This table shows that the main factors affecting computational time are the image size, number of images, and number of identified projected features. The experiments in this study show that when the input data contains 180 images of 1024×1024 pixels, the alignment can be performed in 10 min.

**Table 1:** The test results.

Name	Size (pixel)	Number of images	Number of identified projected features	Number of reliable features	Time (sec.)
Phantom	512×512	180	16	16	102
HeLa1	1024×1024	140	84	6	364
HeLa2	1024×1024	140	89	4	397.5
A1	1024×1024	300	86	3	886
A2	1024×1024	150	85	4	393
A3	1024×1024	140	104	72	518
A4	1024×1024	320	64	8	712
A5	1024×1024	140	121	4	454.5
A6	1024×1024	280	80	8	784.5
A7	1024×1024	140	103	4	406
A8	1024×1024	280	26	3	590
B1	1024×1024	270	0	0	477
B2	1024×1024	300	0	0	490

The run time statistics were obtained by using a MacBook Pro, Intel i7 2.2 GHz, 8 GB main memory, and running Mac OS X 10.8.

## Methods

### Sample Preparation and Image Acquisition

HeLa cells were used in this study. The cells were grown on Kapton film, and endocytosis [16] was used to stain HeLa cells by absorbing gold nanoparticles of 250 *µ*M (micromolar). The cells were then fixed in a container using a mixture of paraformaldehyde and glutaraldehyde[17].

The synchrotron microscope used in this study was built at the National Synchrotron Radiation Research Center, Hsinchu, Taiwan. The CCD size is 2048×2048 pixels, and the field of view is 24 *µ*m. Each projection image was taken after a 1° rotation. To prevent the object holder from becoming perceptible (occurring when the object holder is nearly parallel to the X-ray), the range of the rotation angle of the object holder was ±70°. Only 140 projection images were acquired. The size of each image was 1024×1024 pixels, and the pixel size was 11.78 nm. In this study, the exposure time of each image was 1 second.

### The Alignment Method

A projected feature point is a pronounced mark in an X-ray projection image. Alignment is accomplished by first aligning the projected feature points in the vertical direction and then in the horizontal direction. The projected feature points should be maintained in the vertical direction. Thus, vertically aligning the projected feature points in the second image to the previous image is sufficient. However, the location of the feature points in the horizontal direction varies among projection images. Calculating the horizontal location of the feature points is a more difficult task than alignment in the vertical direction. The following subsections describe these steps.

#### Vertical Direction Alignment

For each pair of projection images, the sum of the intensity values on each row is calculated. The sums of the rows form histograms that should be similar in a pair of consecutive images. The vertical displacement can be calculated by minimizing the difference between the histograms.

Given an *N*×*M* image 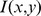, 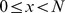, 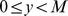 and 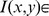
[0, 1]. The vertical histogram 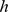 is calculated by 
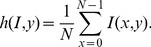
(1)


Assume that 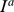 is the unaligned image and 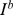 is the reference image. The vertical correction of 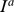 is 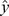, which can be estimated by 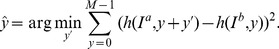
(2)


To achieve the most favorable results, the image is preprocessed to enhance the features. In this experiment, the estimated correction is more accurate when the images are enhanced by applying the edge detection method[18].

#### Horizontal Direction Calibration

The horizontal calibration is based on the projected feature points forming a sine-shaped locus in the 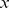-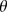 coordinate system. This calibration involves three steps: detecting projected feature points, matching projected feature points to construct a set of loci from the matched projected feature points, and fitting the loci to sine curves to adjust the horizontal displacement of images.

#### Detecting Projected Feature Points

Feature point extraction is a fundamental step in image stitching, object recognition, and feature-based image alignment [19]. Researchers have proposed many feature detection methods. The corner detection method proposed by Harris and Stephen [20] is commonly used to extract corner-shape regions in an image. To achieve scale invariance, Kadir and Brady [21] selected the salient region from the image scale-space as the feature that possesses the maximum entropy. Lowe [22] proposed the scale-invariant feature transform (SIFT) algorithm to select local extrema from the differences of a Gaussian (DoG) pyramid in an image. The SIFT algorithm uses the gradient location-orientation histogram as a feature descriptor to achieve rotation invariance and illumination invariance. Researchers have proposed several improved versions of the SIFT algorithm. Bay et al. used the Haar wavelet to expedite feature detection [23]. Rady et al. proposed entropy-based feature detection [24], and Suri et al. combined mutual information alignment with the SIFT algorithm [25].

In this study, a modified SIFT algorithm was employed to extract automatically the projected feature points contained in X-ray images. The typical SIFT implementation involves describing a feature according to its location, size, the orientation of the sampling region, and the image gradient histogram in the sampling region. Because the proposed method matches the projected feature points in two X-ray images based on mutual information [26]–[28], each projected feature point in this study contained the entropy of the sampling region rather than the image gradient histogram. To reduce the noise and the number of low-contrast projected feature points, the entropy of each selected projected feature point must exceed a given threshold. The experiments in this study entailed setting a threshold between 0.5 and 1.0. Because the features in the objects are gold nanoparticles, the size and orientation of the sampling region were fixed in this implementation.

#### Matching Projected Feature Points

Let 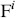, 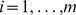 be the sets of projected feature points in 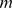 projection images. The projected feature points are classified into 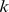 groups. In the ideal case, each group is the set of projected feature points, which are the projections of a feature (i.e., gold nanoparticle) in the object from various angles. Because the rotation angle of the object is small, the projected feature points are in proximity and have similar mutual information in two consecutive images. However, the distance between the two matched projected feature points depends on the distance between the feature and the rotation axis of the object. This means that an affine transform cannot match the projected feature points in two images. Therefore, this study presents a greedy method for classifying the projected feature points. For each pair of images, the random sample consensus (RANSAC) method[29] was first applied to compute an initial alignment of the two images, and a tracking method was then employed to match the projected feature points in the next image.

Several feature tracking methods are available [19]. The proposed method is designed based on the Shafique and Shah's method [30], which is a greedy algorithm for tracking moving objects in videos, and the Tang and Tao's method [31], which integrates the hidden Markov model to eliminate unreliable matches.

Given the projected feature points sets 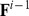 and 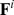, the RANSAC method was applied to compute a translation matrix 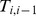, so that a sufficient number of projected feature points 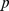 and 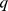 in 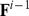 and 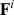 respectively, 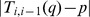 is less than a given threshold 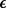. Applying translation matrices 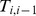, 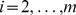 to the consecutive images achieves the initial alignment of the 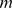 projection images. All of the images are aligned based on the first image.

Given the initially aligned projected feature points 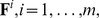 the following procedures yield a set of possible loci of the projected feature points produced by feature points in the object.

**Figure 12:**
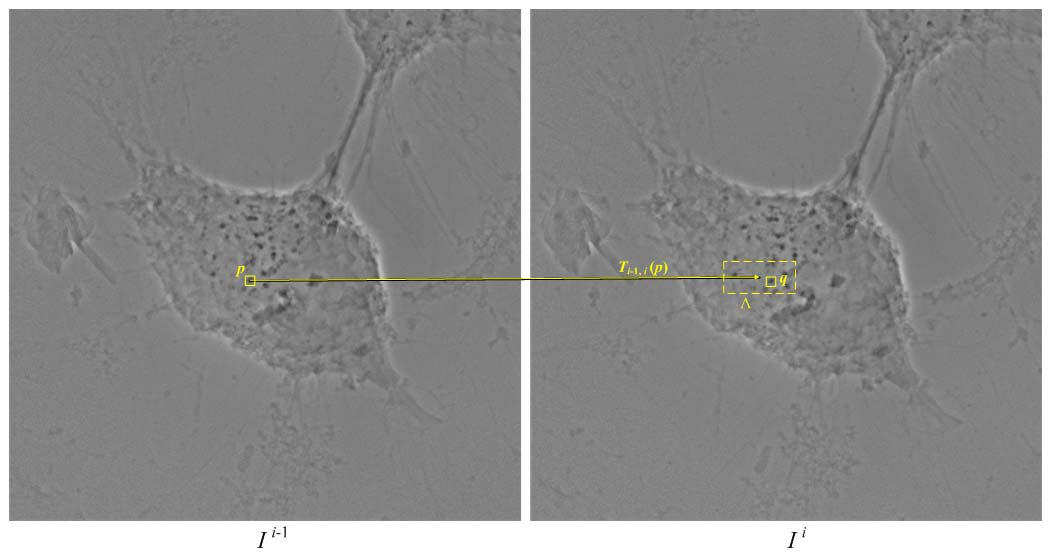
The feature tracking between two X-ray projection images *I^i^* ^−1^ and *I^i^*. For a point *p*, its corresponding projected feature point, 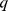, can be searched in the area 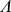 that is determined by applying the affine transformation 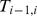 to 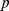.

1. Every projected feature point in 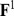 is the starting point of a locus.
2. Iteratively process 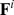, 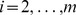;
  - Let 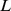 be the set of the loci computed. For each locus 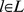, compute 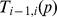 where 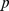 is the final point of 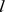 and 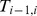 is the inverse of 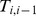. Let 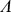 be a region in 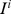 centered at 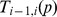. Search in 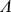 for the projected feature points (Fig. 12). If this region contains only one projected feature point 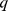, then that point 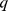 is selected as the final point of 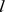. If the region contains more than one projected feature point, then select the 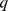 that has the greatest 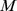, where 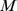 is the average entropy of 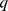 and the previous 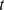 points on 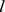. If 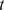 is greater than 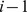, then 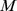 is the average entropy of 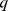 and the points of the entire locus 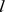.
  - If 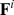 contains unmatched projected feature points, then each of these points creates a new locus.
3. Reverse the image orders and repeat Step 2, but do not include 2b to backtrack all loci.

The X-ray images used in this study measured 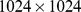 pixels, and 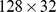 pixels was the size of search region 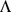, and five previous points for 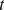.

Because two loci could intersect (i.e., two projected feature points on two loci could overlap or be extremely close), the average entropy must be computed to select the best-matching projected feature point in Step 2a. In this step, some projected feature points with a high entropy in 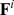 are not included in any locus. These significant projected feature points should not be disregarded, and Step 2b entails creating a new locus for each of them.

After Step 2, the forward feature tracking required to construct the set of loci is complete. To verify the correctness of the loci and complete the loci added in the Step 2b, backtrack all loci in the final step.

### Horizontal Displacement Estimation

Let the set of 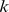 loci collected in the previous step be 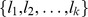. Each locus, 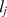, 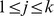, consists of 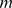 projected feature points, 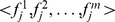. These projected feature points are expected to be the projections of a feature, 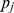, in the object from various angles. Assume that these projected feature points are the projections from 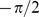 and 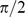. Recall that 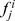 is not a point, but a rectangular box. Take the 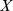-coordinates of the center of the rectangular boxes, 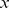, and transform the 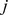 to 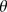, the direction of the projections. Then, draw 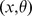 for all 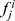 in the 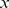-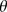 coordinate system. The locus of 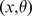 corresponds to a sine curve, (i.e., the sinogram). Given a set of loci of features, the horizontal alignment is conducted by fitting the curves to a set of sine curves and then by computing the deviations.

Consider a locus, 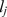, and assume that the projected feature points on the locus are the projections of the feature point 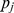. This feature point 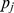 can be expressed as 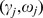, where 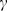 is the radial coordinate and 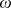 is the angular coordinate. According to the Radon transform [8], the corresponding horizontal position 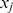 in the 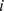 th image (rotation angle 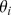), 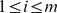, can be written as 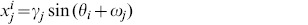
(3)

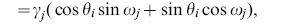
(4)or in matrix form as 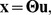
where 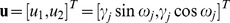, 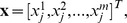 and 
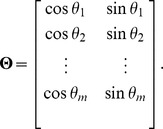


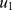
 and 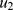 can be solved by the least-squares method, 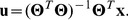
(5)


Finally 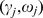, 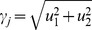
(6)and 
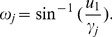
(7)


The horizontal displacement 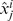 is estimated by 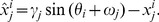
(8)


Because both 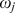 and 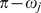 are solutions to (7), choose the one that minimizes the sum of errors, 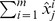. To determine the horizontal displacement 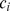 for the 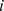 th image from 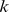 feature loci, use the average of the 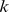 horizontal corrections: 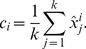
(9)


Some loci are unreliable because of noises, out-of-view projected feature points, or bad projected feature points matching. These loci should be removed. For each locus 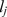, adjust the points on 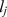 based on the estimated 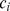, 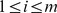. 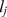 is unreliable if the absolute peak distance between a point on the adjusted 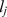 and its best-fitting sine wave is greater than a given threshold 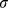. If there are no unreliable loci, stop the algorithm and output the aligned results. Otherwise, remove the unreliable loci and repeat the horizontal displacement estimation algorithm.

## Discussion

This paper presents an image alignment method for X-ray images produced by synchrotron-radiation microscopy. The proposed method enables reconstructing the 3D volume of small objects. The proposed method identifies projected feature points and classifies the projected feature points into a set of loci. The key idea of this method is that the projection of a point in the object should be a sine wave in the 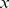-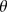 coordinate system. Thus, fitting the set of loci to a set of sine waves can compute the parameters required for alignment.

The proposed method was applied to 12 cases of HeLa cells, and only two of the 12 could not be reconstructed. Compared with the available software systems, SPIDER and Xradia, they could respectively construct three and five cells. Neither SPIDER nor Xradia could construct the two unsuccessful cases of the proposed method. The main reason for the unsuccessful cases is the insufficient number of projected feature points in the X-ray images. The most crucial factor affecting the performance of the proposed method is the number of reliable projected feature points. If there are enough reliable projected feature points, even if the projected feature points are not in the field of view in some projections, the method still works well because it also considers the set of partial loci. The proposed method performs most favorably if the features that produce the projected feature points are close to the rotational axis. Carefully adjusting the rotation axis before images acquisition can improve the quality of the reconstruction.

Considering the shape of the projected feature points, aside from particle objects, the proposed method can manage any shape of object if it contains a sufficient number of distinct projected features. For examples, the corners of a square, the two tips of a rod, and the branch points of a tree-structured object can be used as feature points as long as the features do not deform during image acquisition. If the images satisfy these requirements, then the proposed method can successfully align the images.

Table 1 lists the computing time required for the 10 successful reconstructions of HeLa cells. A graphical user interface software system for the Windows system and Mac OS X 10.8 has been developed. The software system can be downloaded from the following URL: http://www.cs.nctu.edu.tw/~chengchc/SCTA or http://goo.gl/s4AMx.

## References

[1] Shenoy G (2003) Basic characteristics of synchrotron radiation. Structural Chemistry 14: 3–14.

[2] Meuli R, Hwu Y, Je JH, Margaritondo G (2004) Synchrotron radiation in radiology: radiology techniques based on synchrotron sources. European Radiology 14, Issue 9: 1550–1560. 10.1007/s00330-004-2361-x 15316744

[3] Hwu Y, Tsai WL, Je JH, Seol SK, Kim B, et al (2004) Synchrotron microangiography with no contrast agent. Physics in Medicine and Biology 49: 501–508. 15005160 10.1088/0031-9155/49/4/002

[4] Lo TN, Chen YT, Chiu CW, Liu CJ, Wu SR, et al (2007) E-beam lithography and electrodeposition fabrication of thick nanostructured devices. Journal of Physics D: Applied Physics 40: 3172–3176.

[5] Hwu Y, Tsai WL, Chang HM, Yeh HI, Hsu PC, et al (2004) Imaging cells and tissues with refractive index radiology. Biophysical Journal 87, Issue 6: 4180–4187. 10.1529/biophysj.103.034991 PMC1304927 15465870

[6] Larabell CN, Gros MAL (2004) X-ray tomography generates 3-d reconstructions of the yeast, saccharomyces cerevisiae, at 60-nm resolution. Molecular Biology of the Cell 15: 957–962. 14699066 10.1091/mbc.E03-07-0522 PMC363052

[7] McDermott G, Gros MAL, Knoechel CG, Uchida M, Larabell CA (2009) Soft x-ray tomography and cryogenic light microscopy: The cool combination in cellular imaging. Trends in Cell Biology 19: 587–595. 19818625 10.1016/j.tcb.2009.08.005 PMC3276488

[8] Natterer F (2001) The Mathematics of Computerized Tomography. SIAM.

[9] Feldkamp LA, Davis LC, Kress JW (1984) Practical cone beam algorithm. Journal of the Optical Society of America 1: 612–619.

[10] Andersen AH, Kak AC (1984) Simultaneous algebraic reconstruction technique (sart): a superior implementation of the art algorithm. Ultrasonic Imaging 6: 81–94. 6548059 10.1177/016173468400600107

[11] Mao Y, Fahimian BP, Osher SJ, Miao J (2010) Development and optimization of regularized tomographic reconstruction algorithms utilizing equally-sloped tomography. IEEE Transaction on Image Processing 19: 1259–1268. 10.1109/TIP.2009.2039660 20051344

[12] Frank J (2006) Three-Dimensional Electron Microscopy of Macromolecular Assemblies: Visualization of Biological Molecules in Their Native State. Oxford University Press.

[13] Shaikh TR, Gao H, Baxter WT, Asturias FJ, Boisset N, et al (2008) Spider image processing for single-particle reconstruction of biological macromolecules from electron micrographs. Nature Protocols 3: 1941–1974. 19180078 10.1038/nprot.2008.156 PMC2737740

[14] Xu F, Mueller K (2005) Accelerating popular tomographic reconstruction algorithms on commodity pc graphics hardware. IEEE Transactions on Nuclear Science 52: 654–663.

[15] Ikits M, Kniss J, Lefohn A, Hansen C (2004) Volume Rendering Techniques, Addison Wesley, chapter 39. GPU Gems: Programming Techniques, Tips, and Tricks for Real-Time Graphics. pp. 667–692.

[16] Chen HH, Chien CC, Petibois C, Wang CL, Chu YS, et al. (2011) Quantitative analysis of nanoparticle internalization in mammalian cells by high resolution x-ray microscopy. Journal of Nanobiotechnology 9.. 10.1186/1477-3155-9-14 PMC3098147 21477355

[17] Chen HH, Chien CC, Petibois C, Wang CL, Chu YS, et al (2011) Quantitative analysis of nanoparticle internalization in mammalian cells by high resolution x-ray microscopy. Journal of Nanobiotechnology 9: 14. 21477355 10.1186/1477-3155-9-14 PMC3098147

[18] Gonzalez RC, Woods RE (2006) Digital Image Processing (3rd Edition). Prentice-Hall, Inc.

[19] Szeliski R (2010) Computer Vision: Algorithms and Applications. Springer.

[20] Harris C, Stephens M (1988) A combined corner and edge detector. In: Proceedings of the 4th Alvey Vision Conference. Manchester.

[21] Kadir T, Brady M (2001) Saliency, scale and image description. International Journal of Computer Vision 45: 83–105.

[22] Lowe DG (2004) Distinctive image features from scale-invariant keypoints. International Journal of Computer Vision 60: 91–110.

[23] Bay H, Tuytelaars T, Gool LV (2006) Surf: Speeded up robust features. In: 9th European Con-ference on Computer Vision. Graz, Austria.

[24] Rady S, Wagner A, Badreddin E (2008) Entropy-based features for robust place recognition. In: IEEE International Conference on Systems, Man and Cybernetics, 2008. Singapore.

[25] Suri S, Schwind P, Reinartz P, Uhl J (2009) Combining mutual information and scale invariant feature transform for fast and robust multisensor sar image registration. In: American Society of Photogrammetry and Remote Sensing. 75th Annual ASPRS Conference. Baltimore, MD, USA.

[26] Viola P (1997) Alignment by maximization of mutual information. International Journal of Computer Vision 24: 137–154.

[27] Pluim JPW, Maintz JBA, Viergever MA (2003) Mutual information based registration of medical images: A survey. IEEE Transactions on Medical Imaging 22: 986–1004. 12906253 10.1109/TMI.2003.815867

[28] Dowson N, Bowden R (2008) Mutual information for lucas-kanade tracking: An inverse compositional formulation. IEEE Transactions on Pattern Analysis and Machine Intelligence 30: 180–185. 18000334 10.1109/TPAMI.2007.70757

[29] Fischler MA, Bolles RC (1981) Random sample consensus: a paradigm for model fitting with applications to image analysis and automated cartography. Communications of the ACM 24, Issue 6: 381–395.

[30] Shafique K, Shah M (2005) A noniterative greedy algorithm for multiframe point correspondence. IEEE Transactions on Pattern Analysis and Machine Intelligence 27: 51–65. 15628268 10.1109/TPAMI.2005.1

[31] Tang F, Tao H (2008) Probabilistic object tracking with dynamic attributed relational feature graph. IEEE Transactions on Circuits and Systems for Video Technology 18: 1064–1074.

